# LncRNA MALAT-1 modulates EGFR-TKI resistance in lung adenocarcinoma cells by downregulating miR-125

**DOI:** 10.1007/s12672-024-01133-7

**Published:** 2024-08-28

**Authors:** Jie Luo, Qiaoya Ren, Xiaoxi Liu, Qian Zheng, Ling Yang, Mi Meng, Hu Ma, Sisi He

**Affiliations:** 1 Department of Oncology, The Second Affiliated Hospital of Zunyi Medical University, Zunyi, China; 2 Zunyi Medical University, Zunyi, China; 3 Department of Pathology, Suining Central Hospital, Suining, Sichuan China

**Keywords:** Lung Adenocarcinoma (LUAD), LncRNA MALAT-1, Epidermal growth factor receptor tyrosine kinase inhibitors (EGFR-TKIs), miR-125, Resistance

## Abstract

Molecular targeted therapy resistance remains a major challenge in treating lung adenocarcinoma (LUAD). The resistance of Epidermal growth factor receptor tyrosine kinase inhibitors (EGFR-TKIs, epidermal growth factor receptor-tyrosine kinase inhibitor) plays a dominant role in molecular targeted therapy. Our previous research demonstrated the role of MALAT-1 (Metastasis-associated lung adenocarcinoma transcript 1) in the formation of Erlotinib-resistant LUAD cells. This study aims to uncover the mechanism of MALAT-1 overexpression in Erlotinib-resistant LUAD cells. The RT^2^ LncRNA PCR array system was used to explore MALAT-1 regulation in Erlotinib-resistant LUAD cells through patient serum analysis. Dual luciferase reporter experiments confirmed the binding between MALAT-1 and miR-125, leading to regulation of miR-125 expression. Functional assays were performed to elucidate the impact of MALAT1 on modulating drug resistance, growth, and Epithelial-mesenchymal transition (EMT, Epithelial-mesenchymal transition) in both parental and Erlotinib-resistant LUAD cells. The investigation unveiled the mechanism underlying the competing endogenous RNA (ceRNA, competing endogenouse RNA) pathway. MALAT1 exerted its regulatory effect on miR-125 as a competing endogenous RNA (ceRNA). Moreover, MALAT1 played a role in modulating the sensitivity of LUAD cells to Erlotinib. Rab25 was identified as the direct target of miR-125 and mediated the functional effects of MALAT1 in Erlotinib-resistant LUAD cells. In conclusion, our study reveals overexpress MALAT-1 cause the drug resistance of EGFR-TKIs in non-small cell lung cancer (NSCLC) through the MALAT-1/miR-125/Rab25 axis. These findings present a potential novel therapeutic target and perspective for the treatment of LUAD.

**Supplementary Information:**

The online version contains supplementary material available at 10.1007/s12672-024-01133-7.

## Introduction

Lung cancer ranks as the most prevalent malignant neoplasm worldwide [1]. China is a significant contributor to the global burden of lung cancer, with an anticipated surge in patients by 2025. It is projected that the number of lung cancer cases in China will surpass one million, solidifying its position as the country with the highest incidence of lung cancer worldwide [2, 3]. It is frequently classified into two main subtypes: non-small cell lung cancer (NSCLC) and small cell lung cancer (SCLC). Non-small cell lung cancer (NSCLC) comprises approximately 85% of all lung cancer cases. In our country, which has a significant burden of lung cancer, it is projected that the number of lung cancer patients will surpass one million by 2025, solidifying our position as the nation with the highest incidence of lung cancer globally [4, 5]. Nevertheless, a significant proportion of lung cancer patients are diagnosed at an advanced stage, precluding them from surgical intervention. Currently, the primary treatment modality remains a comprehensive approach incorporating radiotherapy and chemotherapy [6], and the five-year survival rate is only about 15% [7]. In recent years, molecular targeted therapy has rapidly advanced in the field of oncology due to its high specificity, relevance, minimal side effects, and promising efficacy. This has led to extensive and in-depth research in the field [8]. Molecular targeted therapy aims to identify and target overexpressed molecules within tumor cells. By using specific drugs that precisely intervene with these molecules and their closely associated regulatory counterparts involved in tumor initiation and progression, it effectively inhibits the proliferation, invasion, and metastasis of tumor cells [9, 10], so as to achieve the purpose of tumor treatment. EGFR-TKIs serve as a key class of targeted drugs for lung cancer. They exert their efficacy by effectively inhibiting downstream signaling pathways mediated by the epidermal growth factor receptor (EGFR) [11]. EGFR-TKIs specifically target and act on tumor cells with precision [12]. However, resistance to EGFR-TKIs, including gefitinib, erlotinib, icotinib, and other novel agents, inevitably emerges after an initial response, known as acquired resistance [13, 14].

In recent years, there has been a rising number of studies showing the importance of lncRNAs(Long non-coding RNAs) in controlling the progression and occurrence of cancer [15–17]. The expression of lncRNAs exhibits dynamic variations throughout different stages of growth and development, and their abnormal expression patterns are strongly associated with the development of human malignant tumors [18]. Among them, metastasis-associated lung adenocarcinoma transcript 1 (MALAT1) stands out as a prominent member of the lncRNA family. It spans 6.7 kb in length, is located on chromosome 11q13.1, and lacks protein-coding potential. MALAT-1 was initially identified in 2003 during investigations into the metastasis of early-stage non-small cell lung cancer (NSCLC) [19]. High expression of MALAT1 in diverse tumor types is consistently associated with poor patient survival outcomes [20]. The role of MALAT1 in the development and progression of various forms of cancer is well known. Some of these include breast cancer, lung cancer, and prostate cancer. Its dysregulation is closely associated with the initiation and development of these aggressive tumor types [21–24]. Furthermore, a recent study has reported that MALAT1 exerts its function by downregulating miR-125, thereby promoting the onset and progression of bladder cancer [25]. LncRNAs play a pivotal role in human disease pathogenesis by modulating gene expression through chromatin modification, transcriptional regulation, and post-transcriptional regulation [26–28]. LncRNAs can act as ceRNAs, forming a sponge-like complex with microRNAs to regulate their expression and impact downstream gene expression [29, 30]. In our previous research, we confirmed that miR-125 induces resistance to EGFR-TKIs in lung adenocarcinoma by upregulating its target gene Rab25 through the PI3K/AKT signaling pathway. However, the regulatory role of LncRNA MALAT-1 in miR-125 remains unknown. This study aims to explore the relationship between MALAT-1 and miR-125 and investigate their involvement in molecular targeted therapy and drug resistance mechanisms in lung adenocarcinoma.

By analyzing clinical specimens, we observed higher expression levels of MALAT-1 in EGFR-TKI-resistant patients compared to those receiving pre-treatment. Through bioinformatics analysis using LncBASE, Targetscan, RNA22, and UCSC, we predicted the binding site between MALAT-1 and miR-125. Subsequent double luciferase reporter assays confirmed the interaction between MALAT-1 and miR-125, resulting in the suppression of miR-125 expression. In vitro and in vivo experiments were conducted on parental and erlotinib-resistant LUAD cells to investigate the role and mechanism of MALAT1 in regulating erlotinib resistance. Through these experiments, we unraveled a novel molecular pathway involved in drug resistance in LUAD, shedding light on its mechanism and functional significance.

## Result

### MALAT1 can regulated the expression of miR-125 in LUAD cells

**Fig. 1:**
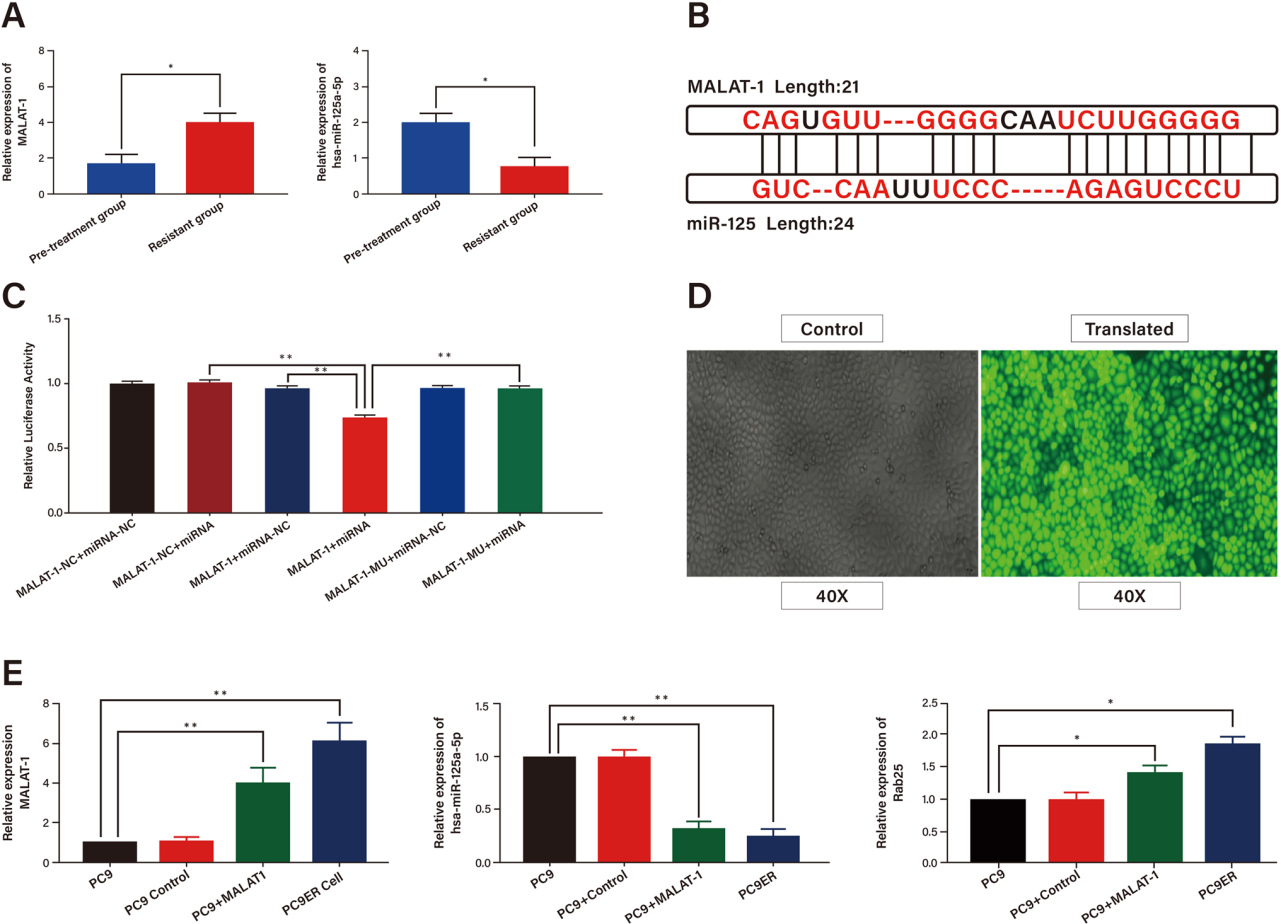
MALAT1 can regulated the expression of miR-125 in LUAD Cells. **A** Relative expression of MALAT-1 and miR-125 in clinical patients before and after drug resistance detected by q-PCR. **B** LncBASE predicts the binding site information of MALAT-1 and miR-125. **C** Double luciferase report experiment verifies that MALAT-1 can directly bind to miR-125. **D** Fluorescence of cells transfected with lentivirus. **E** The relative expression of MALAT-1, miR-125 and Rab25 in PC9, PC9 + Control, PC9 + MALAT-1 and PC9ER cells was detected by q-PCR. *p < *0.05*, **p < *0.01*; n.s., no significance

A total of 88 subjects were enrolled, including 42 males and 46 females, ranging in age from 36 to 77 years old, with an average age of 56 ± 11.4 years old. After the patients signed the informed consent letter, blood samples collected before treatment or within one week after medication were taken as the pre-treatment group. EGFR-TKIs resistance was considered to have occurred, and blood samples were taken again at this time as the post-resistant group. In paired specimens collected from patients before and after developing drug resistance to EGFR-TKIs, our analysis using RT^2^ lncRNA PCR array revealed higher relative expression of MALAT1 in the drug resistance group compared to the pre-treatment group. Conversely, miR-125 exhibited lower expression in the drug resistance group compared to the pre-treatment group, as depicted in Fig. 1A. Furthermore, utilizing bioinformatics tools such as LncBASE and Targetscan, we identified a potential binding site between MALAT-1 and miR-125, as depicted in Fig. 1B. To confirm this interaction, we conducted a dual luciferase reporter assay, demonstrating that MALAT-1 does regulate the expression of miR-125, as depicted in Fig. 1C. MALAT-1 was overexpressed in PC9 cells using SAM dual vector technology, and gene editing was performed using Crispr/Cas9 technology [31, 32] to silence MALAT-1 in PC9ER(Erlotinib-resistant) cells (Fig. 1D). Then, we transfected PC9 + Control PC9 + MALAT-1 PC9ER-shControl and PC9ER-shMALAT-1. qRT-PCR examination revealed that the high expression of miR-125 in PC9ER cells after knocking out MALAT-1, and overexpressed MALAT-1 in PC9 cells leaded to the expression of miR-125 was lower. Rab25, as a target gene of miR-125, was highly expressed in PC9ER and PC9 + MALAT-1 cells, and was low expressed in PC9 and PC9 + Control cells. In short, overexpression of MALAT-1 in PC9 cells led to a decrease in miR-125 expression and an increase in Rab25 expression (Fig. 1E). All these results revealed that MALAT1 might modulate miR-125 expression by acting as a ceRNA.

### MALAT1 enhanced the resistance of LUAD cells by affecting cell proliferation, apoptosis, and cell-cycle distribution

**Fig. 2:**
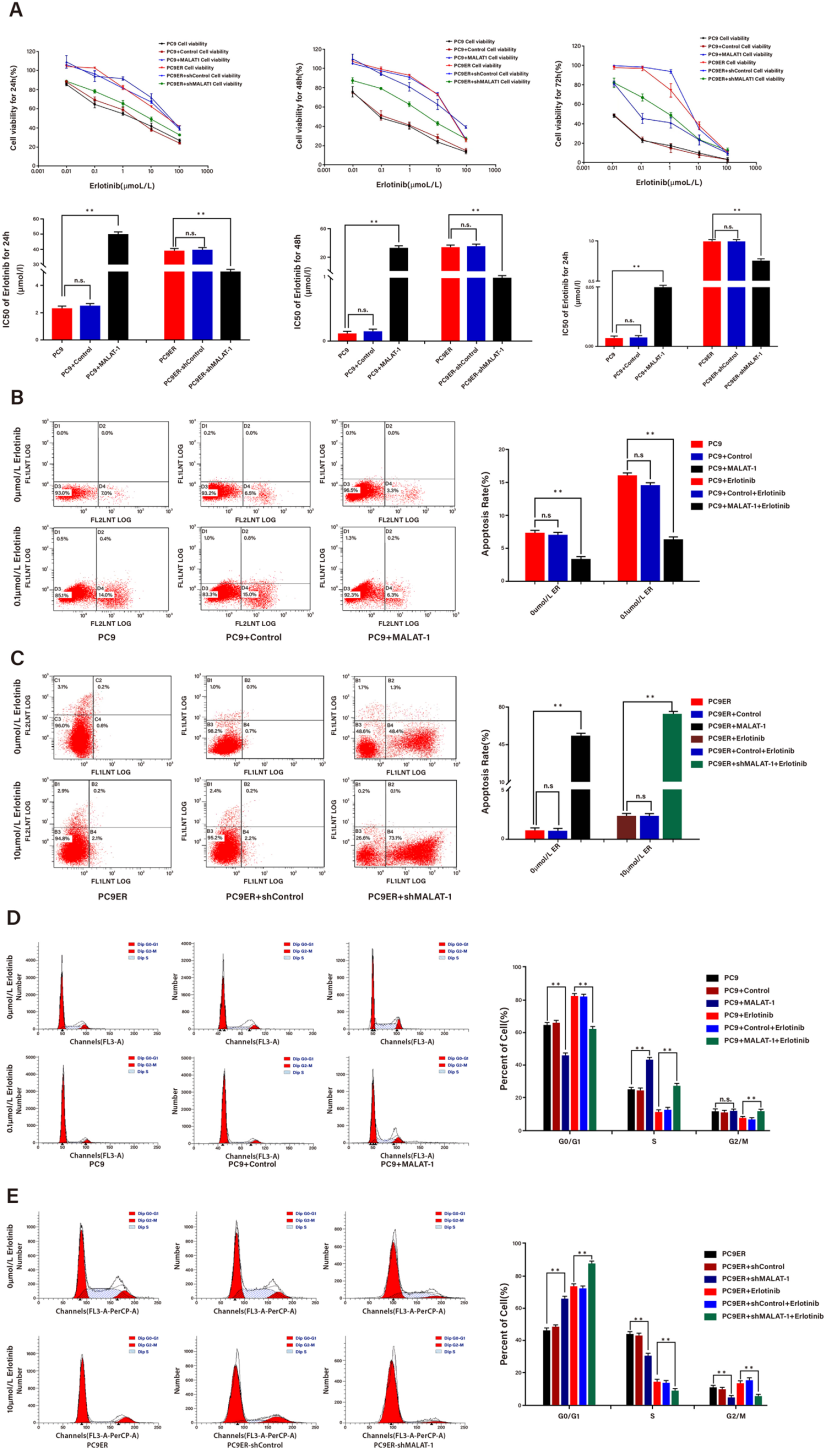
MALAT1 enhanced the resistance of LUAD Cells by affecting cell proliferation, apoptosis, and cell-cycle distribution. **A** CCK-8 experiment has detected the effect of expressing MALAT-1 in parent cells and knocking out MALAT-1 in drug resistant cells on IC50 value. **B** and **C** Flow cytometric analysis of apoptosis in PC9 cells transfected with empty vector or MALAT1 with or without treatment of Etlotinib or in PC9ER cells transfected with shCtrl or shMALAT1 with or without treatment of Etlotinib. **D** and **E** Flow cytometric analysis of cell cycle distribution in indicated cells. *p < *0.05*, **p < *0.01*; n.s., no significance

To investigate the effect of MALAT1 on the resistance of LUAD, we applied cell counting Kit-8 assay to revealed the half maximal inhibitory concentration (IC50) value of PC9ER was higher than that of PC9 at three points in 24, 48 and 72 h. Afterwards, we detected the effect of MALAT-1 overexpression or knockdown on IC50 in parental or Erlotinib-resistant LUAD cells. The results of CCK-8 assays revealed that MALAT1 overexpression significantly increased the IC50 value in parental cells, while MALAT1 knockdown led to the opposite effect in Erlotinib-resistant LUAD cells at three points in 24, 48 and 72 h. There was no significant difference in IC50 between PC9 and PC9ER cells and their corresponding control groups at three points in 24, 48 and 72 h (Fig. 2A). Further, we detected the effects of MALAT1 overexpression or knockdown on the biological processes of parental or Erlotinib-resistant LUAD cells. Flow cytometry analysis was used to determine whether MALAT1 regulates cell proliferation by influencing cell apoptosis and cell cycle distribution with or without Erlotinib. The results showed that overexpression of MALAT1 reduced the apoptosis rate of parental cells, while MALAT1 knockout increased the apoptosis rate of Erlotinib-resistant LUAD cells. There was no significant difference in apoptosis rate between PC9 and PC9ER cells and their corresponding control groups (Fig. 2B and C). Cell cycle experiment demonstrated the overexpression of MALAT1 promotes the cell cycle progression in parental cells, while the knockout of MALAT1 induces the cell cycle arrest in G0/G1 phase of Erlotinib resistant LUAD cells (Fig. 2D and E). These findings indicated that MALAT1 enhances the resistance of PC9 + MALAT-1 and PC9ER cells to Erlotinib, reduces apoptosis and promotes cell cycle progression by increasing cell proliferation.

### MALAT1 was involved in the EMT process of parental and Erlotinib-resistant LUAD cells

**Fig. 3:**
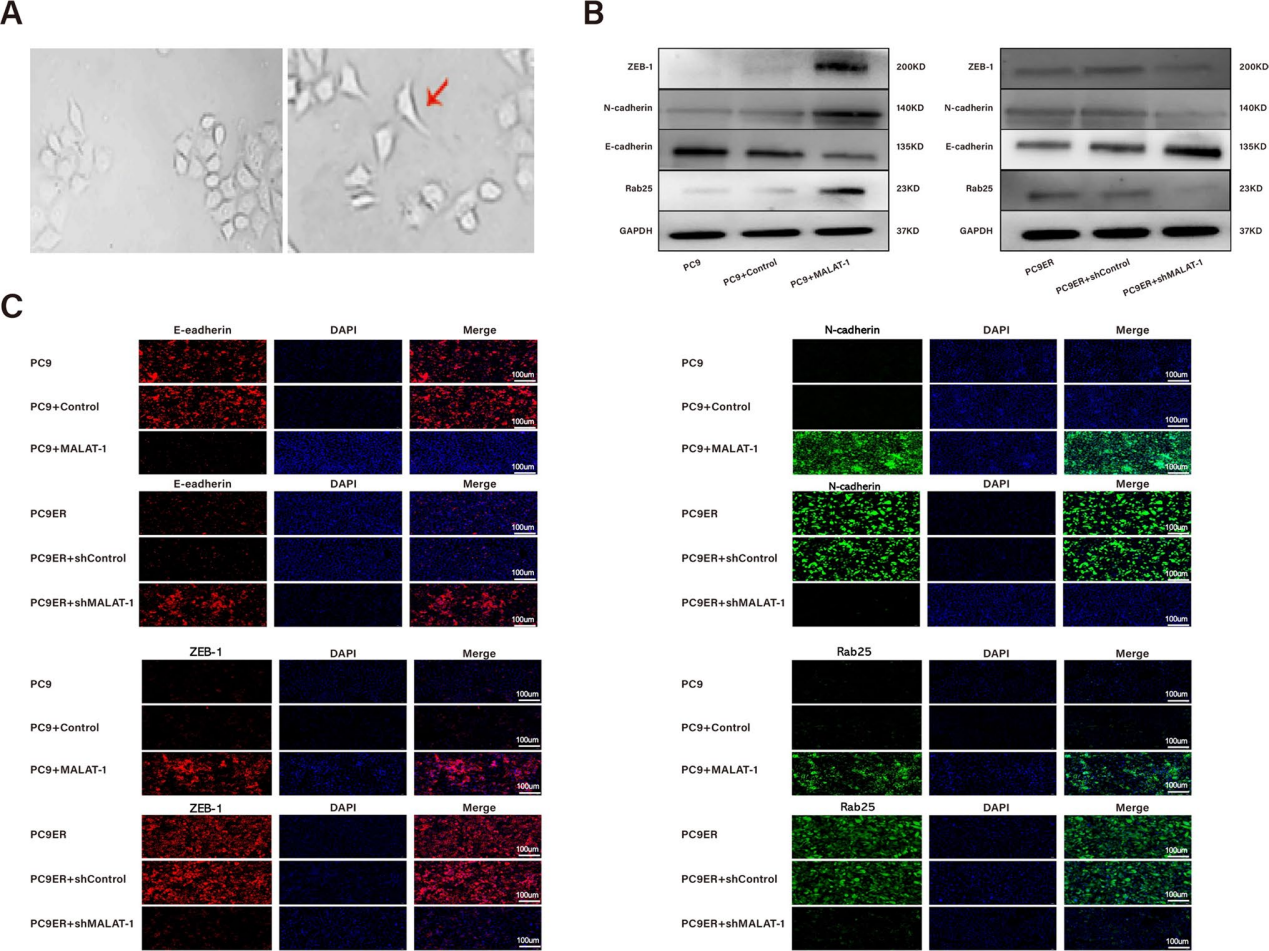
MALAT1 was involved in the EMT process of parental and Erlotinib-resistant LUAD cells. **A** Morphology of PC9 and PC9ER cells. **B** Protein levels of ZEB-1, E-cadherin, N-cadherin and Rab25 in PC9 cells transfected with empty vector or MALAT1 or in PC9ER cells transfected with shCtrl or shMALAT1. **C** The levels of E-cadherin, N-cadherin, ZEB-1 and Rab25 were determined in indicated cells by immunofluorescence staining. Scale bar is 100 mm. *p < *0.05*, **p < *0.01*

The EMT phenotype is an important biological process that has been shown to be closely related to LUAD resistance [33]. In our study, we examined the role of MALAT1 in the progression of EMT. As shown in Fig. 3A, the morphology of the Erlotinib-resistant cells was markedly different from that of the parental cells due to loss of cell polarity, intercellular adhesion, and formation of pseudopodia. To test whether MALAT1 promoted the EMT phenotype of Erlotinib-resistant cells, we used Western Bolt assays to verify the protein levels of Rab25, the downstream targets of MALAT-1, e-cadherin, N-cadherin and ZEB1. The results suggested that MALAT1 overexpression decreased the protein level of epithelial markers (E-cadherin) and increased the level of Rab25 and mesenchymal markers (N-cadherin and ZEB1). In contrast, MALAT1 knockdown in Erlotinib cells led to the opposite result (Fig. 3B). The results of immunofluorescence staining were consistent with those of Western blot (Fig. 3C), which further showed the positive effect and the promotion of MALAT1 to the progression of LUAD EMT.

### Overexpression of MALAT1 accelerated the Growth and Drug resistance of LUAD Cells in Vivo

**Fig. 4:**
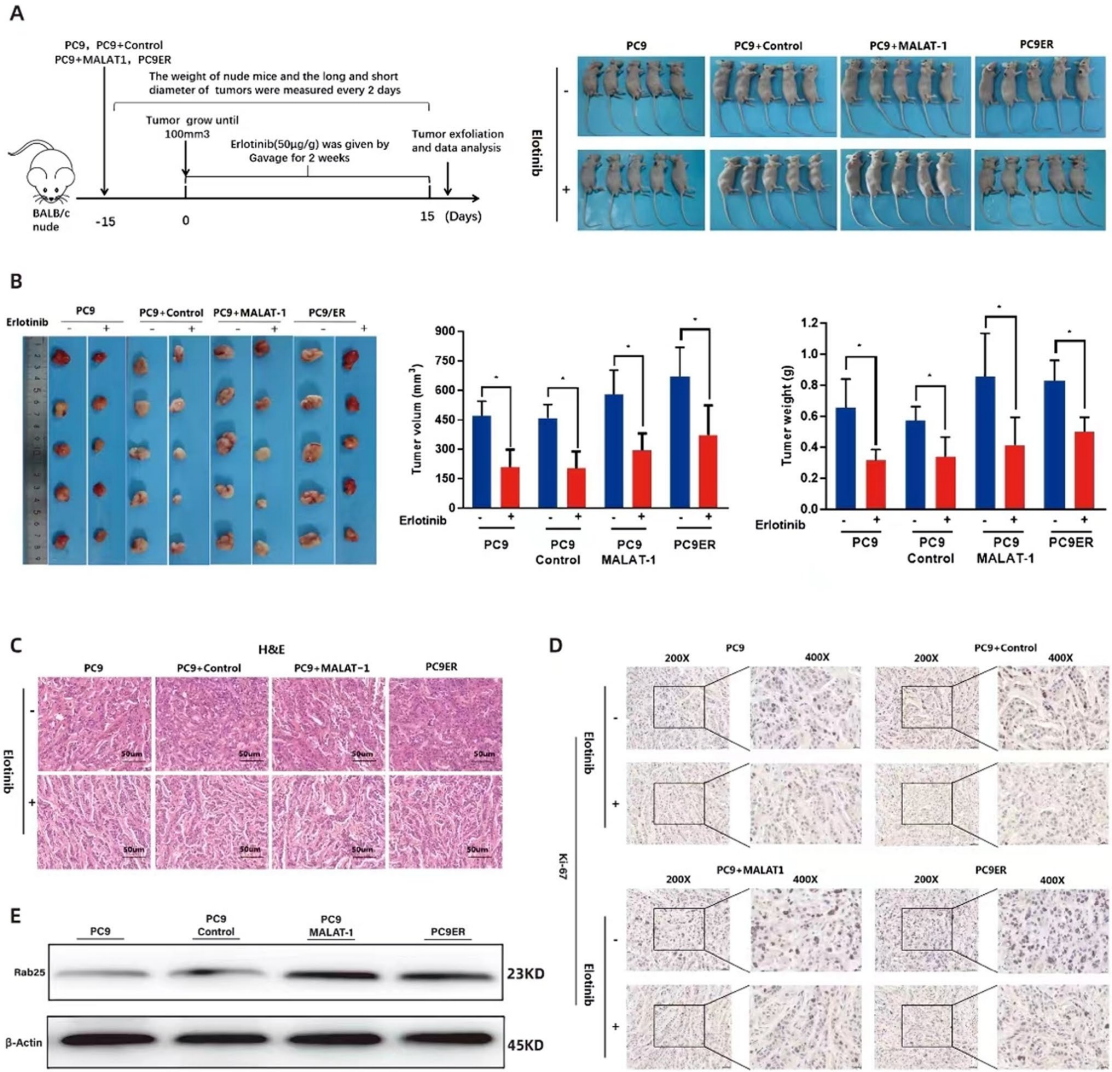
Overexpression of MALAT1 accelerated the Growth and Drug resistance of LUAD Cells in Vivo. **A** Schematic diagram of establishment and treatment of xenograft tumor model and tumor forming nude mice. **B** Tumors derived from LUAD cells transfected with MALAT1 or Control were measured under a condition with Tween 80 or Erlotinib. Tumor volume was measured. Tumor weight was calculated. **C** HE staining of transplanted tumor tissue of nude mice in each group. Scale bar, 50 mm. **D** Immunohistochemical (IHC) staining was performed on tumor sections with anti KI-67 antibody. **E** Western Blot test to verify the expression of Rab25 in each group of transplanted tumors. *p < *0.05*, **p < *0.01*

In order to verify the results of the in vitro experiment, the in vivo experiment was conducted in PC9 cells. First of all, we used stably transfected PC9 + MALAT-1 cells, PC9 + Control cells, PC9 cells and PC9ER cells to establish a nude mouse tumor model (Fig. 4A). Then, cells of each group were injected into nude mice to evaluate the effect of overexpression of MALAT-1 on tumor growth in vivo. The volume and weight of tumor growth in nude mice in PC9ER and PC9 + MALAT-1 groups were larger than those in their parents and control groups (Fig. 4B). HE staining confirmed that the tumorigenic tissue was adenocarcinoma (Fig. 4C). Meanwhile, immunohistochemical experiments confirmed that the positive rate of Ki-67 in nude mice in PC9 + MALAT-1 and PC9ER groups was higher (Fig. 4D). We further used Western Blot experiment to verify the expression of Rab25 in tumor tissues of each group. The relative expression of Rab25 in drug resistant group and overexpression MALAT-1 group were significantly higher than those in parent and control groups (Fig. 4E). The above results indicate that overexpression of MALAT-1 leads to faster tumor growth and higher malignancy.

## Discussion

Currently, the incidence and mortality rates of lung cancer in China remain alarmingly high compared to other malignancies [34]. Furthermore, comprehensive treatment approaches such as radiotherapy and chemotherapy have reached a plateau, necessitating the exploration and development of novel tumor treatment methods. Among these approaches, molecular targeted therapy has emerged as a promising strategy for precise treatment of lung cancer patients, offering new hope in the field. One of the most notable classes of drugs in this realm is EGFR-TKIs [35]. EGFR-TKIs are highly effective in targeting tumors with EGFR mutations by inhibiting downstream signaling pathways mediated by the epidermal growth factor receptor. This treatment approach has demonstrated significant benefits, including tumor size reduction, improved quality of life, and extended median progression-free survival (PFS) of 10–14 months [36].

EGFR-TKIs have demonstrated efficacy in treating patients with NSCLC who have positive EGFR mutations. Both the NCCN and CSCO guidelines recommend the use of EGFR-TKIs as first-line treatment for advanced non-small cell lung cancer patients with EGFR mutations. However, the development of drug resistance is a common occurrence with both first-generation and second-generation EGFR-TKIs after a certain duration of treatment. Drug resistance represents a major obstacle impacting the overall survival (OS) of lung cancer patients [37]. Primary and acquired drug resistance significantly limit the clinical effectiveness of EGFR-TKIs in the treatment of lung cancer [38]. The mechanism underlying primary drug resistance is relatively well understood. Research indicates that EGFR-TKIs can upregulate BIM, a BH3 protein encoded by BIM that promotes apoptosis of mutant cells. Individuals exhibiting primary drug resistance often harbor deletion polymorphisms in intron 2 of the BIM gene, leading to the loss of BH3 function. Consequently, this contributes to primary drug resistance against EGFR-TKIs [39]. In clinical investigations, it has been observed that primary drug resistance constitutes only about 30% of EGFR-TKIs resistance cases, with the majority of patients developing acquired resistance. Apart from the well-known EGFR T790M mutation, small cell transformation, and EGFR bypass activation, there remain several mechanisms that are not fully elucidated. Epithelial-to-mesenchymal transition (EMT) denotes the process by which epithelial cells undergo transdifferentiation into mesenchymal cells under specific physiological and pathological conditions. This concept was initially proposed by Greenburg in 1982 [40]. The onset of EMT is marked by the decrease or loss of epithelial marker proteins such as E-cadherin and catenin, accompanied by elevated expression of mesenchymal marker proteins like N-cadherin, fibronectin, and vimentin. Research indicates that in NSCLC cells resistant to EGFR-TKIs, diminished expression of SFRP5 triggers the activation of the Wnt pathway, facilitating tumor advancement and drug resistance via EMT and Akt2 signaling pathways mediated by TWIST [41]. Additionally, studies have demonstrated that in cells resistant to erlotinib and gefitinib, there is a downregulation in the expression of E-cadherin accompanied by an increase in Vimentin expression. Restoring E-cadherin expression has been shown to moderately enhance the sensitivity of drug-resistant cells to EGFR-TKI [42, 43]. In clinical research, it was found that the primary drug resistance only accounted for about 30% of the resistance to EGFR-TKIs. Most patients had acquired resistance to EGFR-TKIs [44]. Apart from the well-known EGFR T790M mutation, other mechanisms contributing to acquired resistance to EGFR-TKIs include small cell transformation, EGFR bypass activation, MET amplification, ERBB2 amplification, and other factors [45].These mechanisms were not completely clear. Recent research has increasingly highlighted the significant involvement of non-coding RNA in the pathogenesis and progression of various human malignancies [20].

Extensive investigations into non-coding RNA have revealed the significant roles of long non-coding RNA MALAT-1 and short non-coding RNA miR-125 in the initiation, progression, and drug resistance of diverse tumor types [46–50]. In previous research has provided evidence that miR-125, through its regulation of the target gene Rab25 and the PI3K/AKT signaling pathway, contributes to erlotinib resistance in non-small cell lung cancer (NSCLC). In the meantime, we collected serum samples from patients with clinical EGFR mutations, and detected that the expression of MALAT-1 in the group of patients with EGFR-TKIs resistance was higher than that in the group of untreated patients using RT^2^ lncRNA PCR technology. Through comprehensive bioinformatics analysis, we successfully predicted the presence of a binding site between MALAT-1 and miR-125. Subsequently, our experimental findings from the double luciferase reporter assay confirmed that MALAT-1 can indeed suppress the expression of miR-125. These results provide strong evidence for the regulatory interaction between MALAT-1 and miR-125. To explore the mechanism of MALAT-1 involved in drug resistance of lung adenocarcinoma. We knockout MALAT-1 in PC9ER cells by Crispr/Cas9 gene editing technology, overexpressed MALAT-1 in PC9 cells by SAM double vector technology, and successfully constructed PC9 + MALAT-1, PC9 + Control, PC9ER-shMALAT-1 and PC9ER-shControl cells. It was successfully verified by qRT-PCR that the expression of MALAT-1 in PC9 + MALAT-1 and PC9ER cells was significantly higher than PC9 and PC9 + Control cells, The expression level of miR-125 was significantly lower than that of PC9 and PC9 + Control cells, and after overexpression of MALAT-1, the expression level of target gene Rab25 of miR-125 was also significantly increased, which further verified that MALAT-1 could regulate miR-125 and inhibit its expression. Owing to MALAT-1 gene sequence was too long, general gene silencing technology could not be used to knock it out. We used Crispr/Cas9 gene editing technology to knock out MALAT-1 in PC9ER cells, Therefore, it cannot be verified by qRT-PCR experiment [51, 52]. CCK-8 results showed that the IC50 of PC9 + MALAT-1 cells at the three time points of 24, 48, and 72 h of erlotinib effect was significantly increased compared with PC9 and PC9 + Control cells, which significantly reduced their sensitivity to erlotinib. Compared with PC9ER-shMALAT-1 cells and PC9ER-shControl cells, the IC50 of PC9ER-shMALAT-1 cells at the three time points of 24, 48, and 72 h of erlotinib effect was significantly reduced, Increased sensitivity to erlotinib. This assay proves that overexpression of MALAT-1 can augment the drug resistance of lung adenocarcinoma cells to erlotinib, while knockout of MALAT-1 can enhance the drug sensitivity. The results of apoptosis showed that overexpression of MALAT-1 could not only inhibit the apoptosis rate of PC9 cells, but also resist the effect of erlotinib on PC9 cells apoptosis. Knockout of MALAT-1 can increase the apoptosis rate of lung adenocarcinoma cells. Cell cycle results showed that overexpression of MALAT-1 could increase the proliferation activity of lung adenocarcinoma cells, while knockout of MALAT-1 could effectively reduce the proliferation activity of cells. Immunofluorescence and Western Blot experiments showed that compared with PC9 and PC9 + Control cells, PC9 + MALAT-1 cells had significantly lower expression of E-cadherin, while N-cadherin and ZEB-1 had significantly increased expression. Compared with PC9ER and PC9ER shControl cells, PC9ER shMALAT-1 cells had significantly higher expression level of E-cadherin protein, while N-cadherin and ZEB-1 had significantly lower expression level, indicating that overexpression of MALAT-1 could cause EMT transformation in PC9 cells, Knockout of MALAT-1 can reverse this phenomenon, which proves that MALAT-1 can also lead to drug resistance of NSCLC EGFR-TKIs through EMT. The results of immunofluorescence and Western Blot experiments showed that after overexpression of MALAT-1 in PC9 cells, the expression of Rab25 in PC9 + MALAT-1 cells was significantly higher than that in PC9 and PC9 + Control cells. After the deletion of MALAT-1 in PC9ER cells, the expression of Rab25 in PC9ER-shMALAT-1 cells was significantly lower than that in PC9ER and PC9ER-shControl cells. It proved that the regulation of MALAT-1 could lead to the change of Rab25 expression, which led to the drug resistance of NSCLC EGFR-TKIs. After successful establishment of the MALAT-1 overexpression nude mouse model. The results demonstrated that elevated MALAT1 expression enhanced the tumorigenicity and aggressiveness of PC9 cells in xenograft mouse models. It influenced the expression of downstream gene Rab25 and increased drug resistance in lung adenocarcinoma cells. Our study revealed that upregulation of MALAT-1 conferred resistance to Erlotinib in LUAD cells, promoting cell proliferation and inducing an epithelial-mesenchymal transition (EMT) phenotype in PC9 LUAD cells. Conversely, knockout of MALAT-1 in PC9ER cells exhibited the opposite effects. This study investigated that MALAT-1 causes resistance to EGFR-TKIs in LUCA by regulating miR-125 and its downstream target protein Rab25. The experimental findings provided strong evidence for the significant role of MALAT-1 in NSCLC EGFR-TKI resistance.

## Materials and methods

### Cell lines and culture

PC9 lines were obtained from the Tumor Cell Bank of the Chinese Academy of Medical Science (Shanghai, China). Cells were cultivated in RPMI 1640 medium (Hyclon, USA), which was supplemented with 10% fetal bovine serum (Biological Industries), ampicillin (100 U/mL), and streptomycin (100 mg/mL) at 37 ℃ in humidifified air with 5% CO_2_. Erlotinib-resistant LUAD cells (PC9ER) was established from parental PC9 cells and was preserved in 0.1 μmol/L Erlotinib (fifinal concentration).

### Cell transfection

SAM double vector lentivirus with MALAT-1 gene overexpression and Crispr/Cas9 gene editing lentivirus with MALAT-1 gene knockout were designed by Shanghai Jikai Company. SAM double vector lentivirus was transfected into PC9 cells to overexpress MALAT-1 in PC9 cells.

### Construction of stable MALAT-1 knockout, overexpression and corresponding control cell lines

Cas9 lentivirus transfected PC9ER cells to silence MALAT-1 in PC9ER cells. Inoculate PC9 and PC9ER cells in logarithmic growth period into 6-well plate, and the inoculation amount is 5 × 10^4^/mL, the amount of virus inoculated is the number of cells inoculated × The complex number of infection/virus titer was 100. After 12–16 h of infection, purinomycin was added to screen for one week, and then the selected cell line was continuously inoculated into the 6-well plate. After 24 h of culture, lentivirus carrying the target fragment was added. After 12 h of culture, the medium was changed to culture for 72 h. PC9 cells overexpressing MALAT-1 were added with neomycin to screen for another week. PC9ER cells silencing MALAT-1 were cultured for one week, and then observed and verified with fluorescence microscope.

### Bioinformatics analysis

Online bioinformatics analysis found two binding sequences between MALAT1 and miR-125 (http://bicresources.jcbose.ac.in/zhumur/lncrbase). Possible targets of miR-125 are predicted using two bioinformatics prediction tools: TargetScan (http://www.targetscan.org/) and RNA22 (http://www.rnaseqblog.com/rna22-version-2-0-mirnamre-predictions/). MALAT-1 gene sequence comes from the University of California, Santa Cruz (UCSC) (http://genome.ucsc.edu/index.html).

### Double luciferase report experiment analysis

Cells in logarithmic growth phase were made into cell suspension, counted, inoculated in 24 well culture plate (the number of cells was about 10^5^, depending on the size of cell morphology), and cultured in 37 ℃, 5% CO_2_ incubator until the cell fusion degree reached about 60%. Transfection with ROCHE: X-tremegene HP transfection reagent (http://lifescience.roche.com/webapp/wcs/stores/servlet/ProductDisplay?partNumber = 3.5.3.18.1.10). Luciferase detection: 

(http://cn.promega.com/resources/protocols/technical-manuals/0/dual-glo-luciferaseassay-system-protocol).

### qRT-PCR analysis

Total RNA was extracted using TRIzol reagent (Invitrogen) according to the manufacturer's instructions. Use PrimeScript RT kit (Takara, Dalian, China) to reverse transcription according to the manufacturer's instructions. SYBR Prime Script RT-PCR kit (Takara) is used for qRT-PCR according to the manufacturer's scheme. All mRNA expression levels were calculated by using the 2 ^−ΔΔCt^ method and were normalized to GAPDH, U6 or β- Action expression. All measurements were performed three times independently.

### Cell counting Kit-8 chemosensitivity assay

The cells were inoculated into 96 well plates, added with the corresponding concentration of erlotinib, labeled and put into the incubator for culture. Set the culture time of 24, 48 and 72 h according to the experimental needs, take out the 96 well plate, suck out the culture medium in the plate, prepare the CCK-8 detection reagent according to the concentration of 9:1, put it into the incubator for 1–2 h, take out the 96 well plate and put it into the microplate analyzer to detect the OD value of cells at 450 nm wavelength, calculate the cell vitality and IC50 through the OD value. All experimental procedures were repeated at least three times.

### Immunofluorescence assay

Cells were seeded on glass coverslips in 6-well plates, fixed in 4% formaldehyde solution (Solarbio, Beijing, China), and permeabilized with 0.5% Triton X-100/PBS (Servicebio, Wuhan, China). Cells were sealed with 5% BSA/PBS (Servicebio, Wuhan, China) for 1 h at room temperature and then were incubated with primary antibodies (E-cadherin,N-cadherin,ZEB1 and Rab25) (Cell Signaling,American) at 4 ℃ overnight, followed by incubation with fluorescent dye-conjugated secondary antibody (Servicebio, Wuhan, China) for 1 h. Finally, the cells were stained with DAPI (Servicebio, Wuhan, China), and images were observed under a confocal microscope(TCS SP8 STED, Leica, Wetzlar, Germany). All experiments were conducted independently three times.

### Flow cytometric analysis

Inoculate the exponential growth period into the culture bottle with or without Erlotinib for 24 h, collect the cell suspension in a 15 ml centrifuge tube, centrifuge at 1000 rpm for 5 min. The Binding Buffer solution was used to resuspension the cells for precipitation, and was mixed evenly, and the cell concentration was adjusted. Annexin-V (Becton, Dickinson and Company, USA) was added for apoptosis experiment, PI dye (Becton, Dickinson and Company, USA) was added for cell cycle experiment, cells were collected, fixed in 70% ethanol at 4 ℃ for 16 h, and negative control group was set. The results were detected and analyzed by flow cytometry within 1 h. All experiments were conducted independently three times[53].

### Western blot assay

Protein lysates were separated on 10% SDS-PAGE gels and then transferred onto polyvinylidene difluoride membranes (Roche) via electrophoresis. Protein loading was assessed using mouse anti-GAPDH monoclonal antibody. The membranes were incubated with 10% skim milk in TBST at room temperature for 2 h, washed, and then probed overnight at 4 °C with antibodies against ZEB1, E-cadherin, N-cadherin, Rab25, and GAPDH. Following this, the membranes underwent incubation with a secondary antibody conjugated to horseradish peroxidase for 2 h at room temperature. Protein detection was carried out using an enhanced chemiluminescence system and visualized by exposure to X-ray film. All antibodies were procured from Abcam (Cell Signaling Technology, USA). Each sample was subjected to the experiment three times for validation.

### Xenograft model

All BALB/c male nude mice (4–6 weeks old) were purchased from Beijing HFK Bioscience Co., Ltd (certificate number: SCXK (Jing) 2019–0008). The experimental animals were reared in a specific-pathogen-free (SPF) facility of the Key Laboratory of Basic Pharmacology of Ministry of Education, Zunyi Medical University, at a temperature of 20 ± 2 °C, with lighting from 8:00 in the morning to 8:00 in the evening and free access to food and water. The experimental protocol followed the Chinese Animal Protection and Welfare Guidelines and was approved by the Animal Ethics Committee of Zunyi Medical University (2015-07). Mice were randomly divided into four groups: A group (inject PC9 cells); B group (inject PC9 + Control cells); C group (inject PC9 + MALAT-1 cells); D group (inject PC9ER cells). The subcutaneous xenograft model was established by directly subcutaneously injecting 5 × 10^6^ PC9,PC9 + Control,PC9 + MALAT1,PC9ER cells respectively (n = 10 mice/group) into the right flanks of BALB/c nude mice, which were previously suspended in NS. When the average tumor size reached approximately 100 mm^3^, Erlotinib (Shanghai Tengzhun Biotechnology Co., Ltd) was given through gavage at a concentration of 50 μg/mL and the control group of each were given commensurable 1% of Tween 80 (Solarbio, China); one dose was provided every day, with fourteen total doses. Mouse weight and tumor volume was measured using calipers every 2 days and was calculated by the following formula: (long diameter) × (short diameter)^2^/2. After two weeks of treatment, all mice were killed, and tumors were excised, paraffin embedded and formalin fixed or stored at − 80 °C until further real-time PCR and Western blot analysis. H&E staining and Ki-67 immunostaining analysis were conducted.

### Histopathology

A portion of transplanted tumor was cut and fixed with 10% formaldehyde, dehydrated with ethanol gradients, and embedded in paraffin. Tumor tissue was cut into 4-µm slices with a tissue slicer (model: RM2245), and sections were deparaffinized with xylene, hydrated with gradient ethanol, stained with conventional HE, and observed under upright optical microscope (Olympus,Tokyo, Japan).

### Immunohistochemistry assay (IHC)

The tumor tissue sections embedded in paraffin were deparaffinized and rehydrated before immunohistochemistry (IHC) processing. Antigen retrieval was performed using high pressure in a 0.01 M sodium citrate buffer solution. Following incubation with primary and secondary antibodies, the sections were treated with diaminobenzidine and counterstained using hematoxylin (Solarbio, China). Images were captured using a microscope at 200 × magnification (Nikon, E100). Primary antibodies used for IHC included anti-Ki67 (Bioss, USA) and anti-β-catenin (Bioss, USA).

### Statistical analysis

SPSS v.21.0 software (IBM, Armonk, NY, USA) was applied for statistical analyses. Mean ± SD was used to present experimental results. Student’s t test or one-way ANOVA was used to detect the differences among groups. p values < 0.05 were considered statistically significant.

### Supplementary Information

Additional file 1.

Additional file 2.

## Declarations

### Animal experiment statement

In this study, subcutaneous xenograft tumors in nude mice were implanted within the tumor burden approved by the Zunyi Medical University Review Board (IRB) or Ethics Committee to ensure the ethical compliance and safety of the research. In this statement, we confirm the following limitations regarding tumor size or burden:

Animal Experiment: For animal model studies, this research sets a maximum limit of 100 mm^3^ for tumor volume or burden. This limitation aims to ensure animal welfare and prevent undue suffering. We adhere to the maximum limits for tumor size/burden established by the IRB/Ethics Committee.

In this study, we strictly adhere to the limitations on tumor size/burden established by the IRB/Ethics Committee and ensure that all experimental conditions throughout the study comply with ethical and legal requirements to safeguard the rights and welfare of participants.

However, we have observed that the average size of the tumors indeed exceeds the anticipated maximum limit. We have conducted a thorough examination and analysis of this matter and have recognized several potential factors contributing to this circumstance. Some possible reasons include:

1. Experimental variables: There might be certain variables in our experimental process that were not entirely controlled or accounted for, which could have influenced the growth and size of the tumors.

2. Characteristics of the animal model: Biological differences among individual animals may result in variations in tumor growth rates, leading to the exceeding of the expected size range in certain cases.

3. Impact of experimental conditions: Factors such as laboratory environment, housing conditions, drug treatments, etc., could affect the growth and development of tumors, and these factors may have contributed to the exceeding size of the tumors in our experiment.
