# Supplementary material for: LncRNA MALAT-1 modulates EGFR-TKI resistance in lung adenocarcinoma cells by downregulating miR-125
Source: Discov Oncol. 2024 Aug 28;15:379. doi: 10.1007/s12672-024-01133-7 (PMC11358566; doi:10.1007/s12672-024-01133-7)
Supplement: Supplementary file 1 — Additional file 1. [file 12672_2024_1133_MOESM1_ESM.docx]

**Animal Experiment Statement:**

In this study, subcutaneous xenograft tumors in nude mice were implanted within the tumor burden approved by the Zunyi Medical University Review Board (IRB) or Ethics Committee to ensure the ethical compliance and safety of the research. In this statement, we confirm the following limitations regarding tumor size or burden:

Animal Experiment: For animal model studies, this research sets a maximum limit of 100 mm³ for tumor volume or burden. This limitation aims to ensure animal welfare and prevent undue suffering. We adhere to the maximum limits for tumor size/burden established by the IRB/Ethics Committee.

In this study, we strictly adhere to the limitations on tumor size/burden established by the IRB/Ethics Committee and ensure that all experimental conditions throughout the study comply with ethical and legal requirements to safeguard the rights and welfare of participants.

However, we have observed that the average size of the tumors indeed exceeds the anticipated maximum limit. We have conducted a thorough examination and analysis of this matter and have recognized several potential factors contributing to this circumstance. Some possible reasons include:

1.Experimental variables: There might be certain variables in our experimental process that were not entirely controlled or accounted for, which could have influenced the growth and size of the tumors.

2.Characteristics of the animal model: Biological differences among individual animals may result in variations in tumor growth rates, leading to the exceeding of the expected size range in certain cases.

3.Impact of experimental conditions: Factors such as laboratory environment, housing conditions, drug treatments, etc., could affect the growth and development of tumors, and these factors may have contributed to the exceeding size of the tumors in our experiment.
