# Supplementary figures and images for: LncRNA MALAT-1 modulates EGFR-TKI resistance in lung adenocarcinoma cells by downregulating miR-125
Source: Discov Oncol. 2024 Aug 28;15:379. doi: 10.1007/s12672-024-01133-7 (PMC11358566; doi:10.1007/s12672-024-01133-7)

PC9+MALAT-1 PC9+Control PC9 PC9ERsh-M PC9ER-shC PC9ER

N


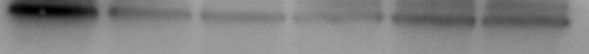


E


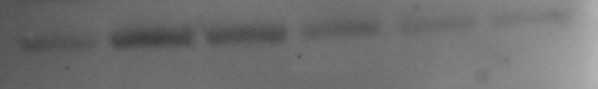


ZEB-1


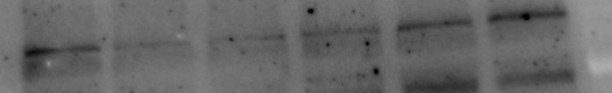


GAPDH


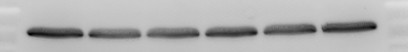


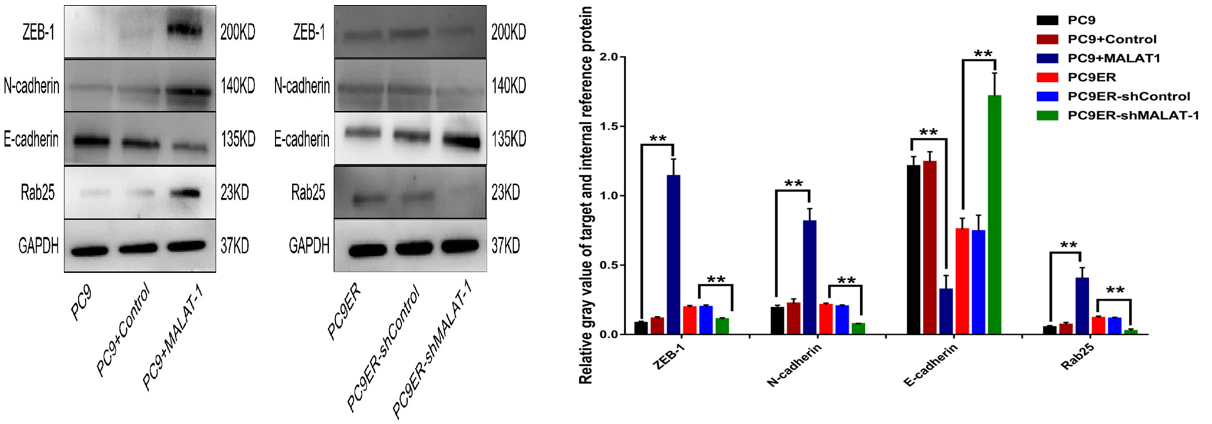

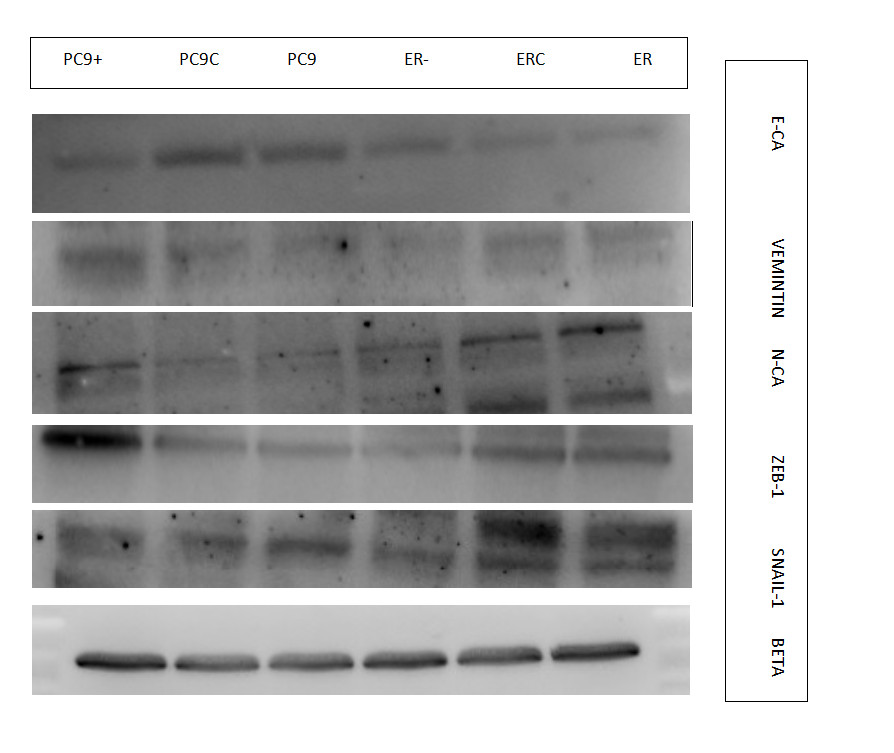

Supplement: Supplementary file 2 — Additional file 2. [file 12672_2024_1133_MOESM2_ESM.doc]
